# The Etiology of Southern Cattle Plague—Texas Fever

**Published:** 1892-08

**Authors:** Frank S. Billings

**Affiliations:** Director of the Patho-Biological Laboratory of the State University of Nebraska


### THE ETIOLOGY OF SOUTHERN CATTLE PLAGUE—TEXAS FEVER.

(*Continued*).

By Frank S. Billings,

*Director of the Patho-Biological Laboratory of the State University of Nebraska.*

O. K.

ROBERT KOCH HELPS THEM OUT.

At the meeting of the International Medical Congress, Berlin, Monday, August 4, 1890, Koch said :

" In numerous places, and indeed in such in which it was least expected, bacteriological investigation has left us completely stranded, especially in the investigation of a number of infectious diseases, which on account of their manifest infectiousness appeared to offer the most favorable conditions for investigation. This has reference, beyond all other disease to the entire group of infectious exanthematous diseases, measles, scarlet, variola and spotted typhus. In no one of these diseases have we been enabled to establish the smallest evidence as to the character of their specific cause. Even in vaccina, which is at the command of every investigator at any time, all our efforts to discover the specific organism thereof, have been in vain. The same is true of rabies.

"We also know nothing of the specific inficiens of the influenza, whooping cough ; the trachoma, yellow fever, the rinderpest and contagious pleuro-pneumonia. I am inclined to the opinion, that in the diseases named, the specific excitor is not to be sought in bacteria, but in disease-producing organisms which belong to entirely different groups of micro-organisms. This conclusion is all the more justified for the reason that lately have been discovered peculiar parasites in the blood of some species of animals, but also in that of men in malarial diseases, which belong in the lowest classes of the animal kingdom, the protozoa. Beyond the simple demonstration of these remarkable and very important parasites we have not gone."

"Ueber Bacteriologische Forschung," *Deutsche Med. Wochenschrift*, 1890, p. 757.

From the very moment that I read those words of the father of modern bacteriological technique, and certainly one of the most noted and painstaking investigators the world has ever produced, I could not help, and cannot yet restrain expressing the utmost astonishment that Robert Koch could have allowed himself to make such an assertion, or come to any such conclusion. Both are absolutely contradicted by every iota of pathogenic experience in reference to the diseases mentioned by Robert Koch. What correspondence is there in the genesis of the influenza, whooping cough, scarlet, measles, variola, spotted typhus, the rinderpest and contagious pleuro-pneumonia, with a malarial disease? They are, one and all, endogenous, obligatory parasitic diseases, while all malarial diseases are paradigmosis of bone, exogenous, facultative parasitic diseases. Where is the danger of transmission in any natural way of specific malarial diseases from diseased to healthy individuals? The diseased individual is as harmless to others as a cooing-dove. Whoever heard of fever and ague being transmitted by cohabitation? Whoever heard of house-quarantine, of burning all the clothes, or scalding or severe germicidal measures, of shutting up school houses, as in the case of the diseases named in man, or of calling out the military, or absolute slaughter as in rinderpest and contagious pleuro-pneumonia in cattle being resorted to in any disease of malarial character? These diseases are specically "contagious" in the ancient hygienic and common sense view, equally scientific of the origin of diseases. *Without the presence of a diseased individual, or something directly from such, no danger.* Koch's a priori hypothesis is no more fortunate as regards the yellow fever, and diseases of that class, diseases of local origin, exogenous facultative parasitic, but in which the diseased individual can transport the specific inficiens if traveling or moved when ill, to other localities and infect them, whereby persons in those localities, if they offer favorable conditions to the continued development of the germ and the retention of its virulence, may also become infected and acquire a disease foreign to their clime. *Hence quarantine regulations in these diseases also.* So far as the experience of the centuries go, and they cannot be passed over as worthless, no malarial disease has ever been transportable in any such manner, and no such restrictions on the movements of persons from malarial districts have ever been advocated. Even though the specificance of fever and ague has been generally admitted to have been discovered by Laveran and others, still all attempts to transmit the disease to healthy organisms have failed. I admit this does not amount to anything until the tranmission to human beings has been tried. On the other hand it is now pretty certain that the specific-causal agent in two of the diseases named by Koch, influenza and measles, has been discovered, and there are rumors in the air of the discovery of a similar agent in variola and vaccina, one of which I have some knowledge of and think to be reliable.

It has always seemed to me that a person of Koch's great experience in bacteriological investigation, and one who certainly knows, as well as any man, of the great difficulty, at times, of the investigator's supplying artificial media and conditions exactly conformable to those offered by the animal organism to the life of specific inficientis, would have at once seen that in this fact was to be sought the cause of the failures he has mentioned, and that any such a priori hypothesis as he then made regarding their origin was absolutely without a particle of evidence in its favor. Even blood serum, when drawn from the body, most carefully and successfully separated and sterile, though uncoagulated, is no longer necessarily a natural nutrative medium, though the changes thus produced in it are not of so radical a nature as to interfere with the extra-organisimal development of many germs, though they are to others, and that is why we have failed to cultivate them and prove their specific infectious disease-producing qualities.

### THE RELATION OF TICKS TO TEXAS CATTLE FEVER.

Sixth and Seventh Reports Bureau of Animal Industry, 1889, 1890. Revised 1891.

While the investigations into the nature of this disease were going on, other equally important work was being carried on at the Experiment Station on the external characters of the disease.

It is well known to those who have come in contact with southern cattle in summer, that they are infested with the so-called cattle tick, a pest belonging to the class Arachnida and to the family Ixodidæ. These ticks are carried north with cattle during the warm season of the year. When fully matured they drop off from the southern animals, lay their eggs on the ground, and perish. The young ticks are hatched within fifteen to thirty days after the eggs are laid, and at once get upon the cattle, where they become mature within twenty to thirty days, to again drop off, lay their eggs, and die. This process goes on continually until the cold weather comes.

At various times and in different parts of the country it has been suggested that the ticks were the cause of Texas fever in northern cattle. This inference was undoubtedly suggested by the fact that nearly all cattle that die of Texas fever, are observed to have these ticks, of various sizes, attached to the skin. Moreover, the disease only makes its appearance after the young ticks have attached themselves to cattle. Though this was purely a *post hoc propter hoc* inference, it was, nevertheless, true, as the experiments to be recorded will amply prove.

During the summer of 1889, Dr. F. L. Kilborne, in arranging the various inclosures at the Experiment Station for the exposure of native cattle to the infection of Texas fever, conceived the happy idea of testing this popular theory of the relation of ticks to the disease. This he did by placing southern (North Carolina) cattle with native cattle, in the same enclosure, and picking the ticks from the southern stock as soon as they had grown large enough to be detected on the skin. This prevented any ticks from maturing and infecting the pasture with the eggs, and hence prevented any ticks from infecting native cattle subsequently. At the same time, in another enclosure, the ticks were left on the southern cattle. The natives in the latter field died of Texas fever, those in the former did not show any signs of the disease.

Another experiment was made in September in the same manner by preparing three fields, one with southern cattle and ticks, a second with southern cattle, from which the ticks were removed, and a third, over which only adult ticks had been scattered. The result was equally positive. In the first field no natives died, but careful examination of the blood by the writer showed Texas fever in an unmistakable manner. In the "tick" field one animal died of Texas fever, and the examination of the blood showed that most other natives in the field were sick. In the third field, containing southern cattle without ticks, no disease could be detected.

These two tests pointed directly to ticks as being in some way the cause of Texas fever. At the same time it was thought best to confirm these results by further experiments during the present year before other agencies could be eliminated. The immediate inference was that the ticks infect the pastures, and that in some unexplained manner the infection finds its way into the body of susceptible cattle. The preliminary conclusions deducible from the work of 1888 and 1889 can be formulated as follows :

(1). Texas fever is a disease not caused by bacteria. Its nature cannot be understood by supposing a simple transfer of bacteria from southern cattle to pastures, and from pastures to northern cattle.

(2). The cause is very probably a protozoön, with a more complex life history, living for a time within the red corpuscles of infected animals.

(3). Southern cattle without ticks cannot infect a pasture.

(4). Ticks alone scattered on a pasture will produce the disease.

The work of 1890 was planned to confirm or refute these preliminary conclusions and to furnish additional information.

The fields were arranged as before. One contained North Carolina cattle with ticks, a second Texas cattle with ticks, a third North Carolina cattle without ticks, a fourth ticks only, and a fifth, soil from the pastures of infected North Carolina farms. Other fields were also laid out to test questions which need not engage our attention in this brief survey.

The results confirmed those of last year. The first animal to die was in the "tick" field containing no southern cattle. No disease appeared in the soil field. Unfortunately, owing to the limited space of ground at our disposal, and its barren, rolling character, ticks or eggs were washed, during the very heavy rains of the summer, from the tick field into the field containing northern cattle without ticks, although a wide lane intervened. The natives in this field thereupon all died with Texas fever. At the autopsy of these cases ticks were found attached to their skin in abundance.

The disease caused by Texas cattle could not be distinguished in character from that which was produced by North Carolina cattle. These results similarly pointed to ticks as the cause. The precise manner in which they caused the disease was by no means clear, however. The theory which seemed for a time most acceptable was that the adult ticks, as they dropped off, infected the pastures with germs which they had taken in with the blood of southern cattle, and that the germs were introduced into the body of northern cattle with the food. At the same time no parasite could be detected in the blood of southern cattle examined at various times, on which fact I would lay no great stress, however. Of more importance is the peculiarity which is exhibited by this disease in its period of incubation, as it may be provisionally denominated, and which is opposed to this theory. Thus, when native and southern cattle are placed on the same pasture at the same time, it will take from forty to sixty days for the disease to appear. After the disease has once shown itself, fresh animals placed on the same pasture may die, according to our experience, within thirteen days after the beginning of the exposure. We might say that the virus has "to ripen" on the pasture, which takes nearly two months, depending on meteorological conditions. When once "ripened" this virus does its deadly work within two or three weeks. This explanation, however, would be merely formulating our ignorance concerning the true nature of the infectious principal.

To the writer there seemed to be but one inference drawn from the facts, and that is that the presence of young ticks is in some way directly associated with the appearance of the disease. It requires from forty to sixty days for the matured ticks to drop from the southern cattle and the eggs laid by them to develop into young ticks. After that period young ticks are present on the pastures until they are destroyed by the cold, or until the cold interferes with the development of the embryo in the egg. In other words, the period of incubation of the disease is explained without any difficulty by the life history of the tick.

The question was solved, experimentally, in the following manner: Eggs laid by ticks sent from North Carolina were placed on dried leaves in a dish partly filled with moist soil, and kept in the laboratory until the young emerged from the egg. The period of incubation depends entirely upon the relative amount of heat, and has varied from fifteen days in midsummer to forty days in November, when the rooms of the laboratory became cold at night (50 to 60 degrees F.). These ticks were placed on four different animals of different ages, kept away from any infected enclosures. Two were placed in stalls, one of them on an adjoining farm, and two were allowed to stay in a patch of woodland with healthy cattle. Of these four, two died of Texas fever, as determined by careful post-mortem examination. One of them was in the stall away from the station, the other in the patch of woodland. The other two became very ill; one of them never recovered, but had to be killed later on; the other recovered. In all of them the germs were observed in the blood. The disease possessed the same characters as those observed in cattle in the infected pastures during the summer. There was an elevation of temperature from nine to twelve days after the young ticks were placed on the animals, going as high as 107 degrees F. in one animal. Accompanying the fever a gradual reduction in the number of blood corpuscles was observed. In order to show more conclusively the truth of the statements made, a few brief notes from one of the experimental cases is appended:

No. 144.—Cow about eight years old, purchased September 16 from a neighboring farm, and placed among a number of healthy reserve cattle in a piece of woodland at some distance from any infected field.

September 17.—A considerable number of young ticks, hatched in the laboratory from the eggs laid by ticks from North Carolina, placed on this animal.

September 18.—Temperature 101.2° F., 6.3 millions red corpuscles in blood; normal.

September 24.—Another lot of recently hatched ticks placed on the animal.

September 27.—A. M., temperature 104° F.

September 29.—10.45 A. M., temperature 106.2° F., pulse 54, respiration 27, 4.93 millions corpuscles in blood. Ticks abundant on body, especially on inside of thighs. Still quite small.

September 30.—P. M., 107°.

October 1.—P. M., 106.3°.

October 2.—P. M., 104°.

October 3.—Found dead this morning. Seen alive at 6 P. M. yesterday. A large number of ticks on animal, just through second molt. None of them large as yet.

Lungs only partly collapsed; trachea and bronchi filled with foam. Ecchymoses under epicardium of both ventricles of heart, and under endocardium of left ventricle.

Spleen very large, blackish, soft, weighs 4 1-16 lbs. (Normal weight about 2 lbs).

Liver weighs about 12 lbs.; enlarged, yellowish on section. Complete injection of the intra-lobular bile capillaries. Extensive fatty degeneration of liver tissue. Occasional groups of hæmatoidin crystals.

Bile dark, scarcely flows. Density due to large quantity of yellowish flakes and mucus.

Kidneys deeply congested; tubules contain much yellowish pigment. Urine in bladder of a deep, port wine color, barely translucent in small test-tube; alkaline; specific gravity 1015; no sediment; albuminous precipitate very abundant (.6 per cent. according to Eabach). Heavy flocculent precipitate when acetic acid added (hæmoglobin).

In preparations of blood from the heart, of liver, spleen, and kidneys, a small number of corpuscles contain parasites, in contracted state, from 1.5 to 2. micromillimetres in diameter. In the blood, spleen, liver, and kidney preparations, a moderate number of large bacilli of post-mortem growth. (These bacilli are invariably present when the animal dies early in the night and is not examined until next day. They are never found in animals killed in a dying condition. They occur in other diseases under similar conditions.)

These brief notes demonstrate that Texas fever can be produced by placing young ticks on cattle, and that the disease can not be due to any abstraction of blood, for the ticks were still quite small, and had scarcely begun to draw blood on a large scale. Moreover, the corpuscles perished *in the body*, as is shown by the coloring matter in the urine, by the thick bile, and the presence of pigment in the liver and kidneys. No disease appeared among the other cattle in the same inclosure.

While the nature of the Texas fever is by no means made clear as yet, we are able to affirm that ticks can produce it. Whether the disease can be transmitted by any other agency must be decided by future investigations. Meanwhile the evidence accumulated thus far, seems to favor very strongly the dictum—*no ticks, no Texas fever.*

So particularly attractive and so valuable a scientific contribution did this last appear, that it received a lengthy editorial notice in the London *Lancet* of April 2, 1892, which was copied into the *Veterinary Journal*, of London, for May, 1892, from which the following is taken:

"IXODES AS A CAUSE OF INFECTION IN CATTLE.

"For many years a mysterious disease has affected cattle in that part of the American continent which borders on the Mexican gulf, and is known in the United States as the splenic, Texas or Spanish fever. It has been observed wherever and whenever bovines from the States on that gulf have been driven northward, and more particularly through the summer months, and is looked upon with the greatest apprehension by cattle breeders. In 1868, for instance, the destruction it wrought among the northern herds with which the traveling Texan droves came in contact, directly or indirectly, and the obscurity that prevailed with regard to the nature of the disease, caused a serious panic, and nearly led to the total suppression of the traffic in cattle between the Gulf and States (which are almost entirely cattle-rearing countries) and the northern States. At that time Prof. Gamgee was in America, and the United States government availed itself of his services in making an investigation of the malady. The report of the commission was published in 1871, and did not throw much light either on the nature of this Texas fever, as it is now generally designated, or its etiology: indeed the latter remained in as great obscurity as ever. The tendency was to regard it as a kind of anthrax or splenic fever, due to the animals becoming affected by something in the soil or water. Their systems becoming charged with poisonous principles that accumulated in the bodies of acclimatised cattle, which enjoyed immunity from the disorder. It was ascertained that those Southern cattle might for some weeks, and probably for as long as three months they would "continue to excrete the deleterious principles which poison the cattle of the States through which the herds are driven on their way North or West." The report also stated that all breeds of cattle in States north of those of the Gulf Coast, without regard to age or sex, if they fed on grass contaminated by Southern droves, were attacked with fever; that it occurred chiefly during the hot months of summer and autumn, and that infected pastures were rendered free from danger by frost, and remained so while fresh droves of Texan or Florida cattle were driven again over them.

"This apparent freedom of the exotic Southern cattle from disease, and their capability, notwithstanding, of disseminating such a fatal infection among native bovines, was an altogether unusual feature in epizootology and could not be accounted for. It is true that the redoubtable cattle plague or rinderpest does not so severely affect the cattle of Southern Russia as it does those in Western countries; yet it kills a good percentage of them, and greatly impairs the condition of those which do not succumb. But here were animals in evidently excellent health, carrying destruction everywhere they went. The principle symptoms in those infected are somewhat rapid rise of fever; from 101, 102, 106 and 107 degrees, at which it remains from four to fifteen days in fatal cases; this condition is soon accompanied by intense debility, that persists until death takes place. A few days before the fatal termination the urine becomes more or less deeply tinged with hæmoglobin, and the destruction of red-blood corpuscles, causing this altered tint of the urine, can be demonstrated by examining the blood obtained from an incision in the skin. In acute cases, a day previous to death, the number of these corpuscles had been found reduced to about one million in a cubic centimetre of blood, the normal number being something like five million and a half. In one case, which recovered, the corpuscles fell to nearly one-half their ordinary number, and a week later they had increased to three millions. This enormous decrease in the red blood corpuscles gives the blood an exceedingly thin and watery appearance; after death it coagulates rapidly and forms clots of unusual size.

The lesions noted in making a necropsy, are chiefly the following: The spleen is very much enlarged, and when incised the tense capsule retracts and exposes a dark red and more or less disorganized pulp, that sometimes pours out as a semi-fluid mass. This engorgement is due chiefly to the presence of an immense number of red corpuscles. The liver is much affected; it is yellowish-brown in color; the tissue is deeply bile-stained, and the smaller canaliculi are often plugged with inspissated bile, that fluid in the gall-bladder being so thick that it scarcely flows, owing to the presence of solids in the form of minute yellow flakes. The kidneys are tinged with hæmoglobin, the connective tissue around them being distended into reddish serum. The other organs are often more or less stained in the same manner, but seldon exhibit any other character.

The heavy losses caused every year by this peculiar fever have compelled the United States Government to take special notice of it, and with the view of thoroughly investigating its pathology, experimental stations have been established at points where it could be best observed. The result of this measure has been very satisfactory. These investigations began in 1889, and attention was more especially directed to the blood. No bacteria were discovered, but there were found, instead, peculiar bodies lodged in the red corpuscles. In the fresh splenic pulp these appeared as round or oval, almost colorless spots, from one-half to nearly two micro-millimetres, from $\frac{1}{50000}$ to $\frac{1}{12000}$ inch in diameter, on the disc of the red corpuscles, and always somewhat excentrically placed. Sometimes there was only one, but quite commonly there were two and very rarely three or four in the same corpuscle. In organs kept in the cold for nearly two weeks, these bodies were still visible, though faintly so, owing to the diffusion of the hæmoglobin around and perhaps into them. They stained with analine dyes as readily as nuclei and bacteria, and retained the stain as tenaciously. They have appeared like deeply stained cocci, about one-half to one micro-millimetre in diameter, situated within the unstained circle of the corpuscle. Besides the spherical forms, ovoid bodies are not uncommon, and these usually occurred in pairs within the red corpuscle; while a still rarer pear-shaped form was met with in stained preparations of the blood. This being rounded at one pole and constructed or filamentous at the other. These almost invariably occurred in pairs, which occupied one corpuscle, and were considered as the result of division of the single body. Another abnormal form was found in the blood. When dried cover glass preparations were stained with Loffer's alkaline methylene blue, and few corpuscles appeared as if their surface had been dusted over with minute specks of coloring matter; but whether these were due to the anæma or whether they belonged to the cycle of the micro-organism, could not be determined. Very few of these bodies were found in the circulating blood, though in one instance a number were detected in this fluid the day before death. Blood from the right ventricle of the heart contained them in enormous numbers, in pairs, within the red corpuscles. They seemed to be filtered out of the spleen and liver, for they might be numerous in one or two of these organs, and rare in blood from the right heart. They appeared to be more abundant in the spleen and liver. In perhaps half the number of cases examined, they were so few in the spleen that they easily escaped the attention of an obscure searching for bacteria. In one case they were so scarce that a number of fields had to be scanned before any were detected, and here the spleen was completely disintegrated, while the urine, which contained much hæmoglobin several days before death, was almost normal at the necropsy, as if the animal had overcome the parasites, but perished from the havoc caused by them.

"It had long been suspected by cattle owners that the appearance of this fever in northern cattle was in some way connected with the ticks (Ixodes Americanus, Lat.: I. bovis Riley) distributed by southern cattle, though this notion was generally scouted by scientific men. However, as every other possible cause of the scourge had evaded detection, it seemed worthy of investigation, and the result now is that indsputable evidence has been obtained that the fever is produced by these ectozoa. Ticks taken from the bodies of southern cattle and placed upon pastures which could have been contaminated in no other way, so infected these grounds that susceptible bovines placed upon them contracted the malady in the same period of time, and were as seriously affected as were the susceptible cattle allowed to consort with those from Texas. Again young ticks were hatched from the eggs of adult ticks picked from southern cattle, placed on the skins of northern animals and induced no disease.

"There are, consequently, two factions in the production of Texas fever, ixodes and the protozoal micro-organism which is located in and destroys the red corpuscles of the blood in the affected animals. Wherever the insect obtains the protozoon is not yet known, but that the latter can be transmitted from one generation of ticks to another, through the ova, has been demonstrated. It is important to know how many generations of ticks these germs can be communicated to without losing their virulence, and whether there is any other means by which they gain access to the blood of cattle than by the punctures made by these vermin. There are evidently ticks which do not bear the micro-organism, as cattle which are susceptible to Texas fever are often badly infected with them without showing any marked sign of bad health. On the other hand, there may be means by which the protozoon gains access to the blood of cattle independently of the agency of ticks ; but it appears from the investigation just reported, that in the great majority of cases cattle are infected by means of the skin parasites. The adult ticks drop from the southern cattle and deposit their ova upon the pastures ; these ova are hatched and the young ticks get on susceptible beasts and engender the disease in them. The young ticks are hatched within from fifteen to thirty days after the eggs are laid, and at once get on the cattle, where they become mature in from twenty to thirty days; they then fall off, lay their eggs and die. This process goes on continually until the cold weather sets in.

The experiments by which these interesting and important results were arrived at, were numerous and conclusive as to the part the ectozoa play in the production of the malady, so that it might be said that, no ticks, no Texas fever. Is it not possible that the Tse-tse fly of Central and South Africa causes the death of certain mammalia by acting as the intermediate bearer of a noxious micro-organism, as the mosquito does with regard to the filaric sanguinis hominis? In no other way, it would be seen, can its deadly action be accounted for.—*The Lancet.*

### A SUMMARY OF THE STATEMENTS OF THE GOVERNMENT INVESTIGATORS.

"It is well known that southern cattle, in summer, are infected with the so-called cattle tick. The ticks are carried north with the cattle during the warm season of the year. When mature they drop off the southern cattle, lay their eggs on the ground, and perish. The young ticks are hatched fifteen or thirty days after the eggs are laid, become mature within twenty or thirty days, to again drop off the cattle, lay their eggs, and die. This continues until cold weather comes. The disease only makes its appearance (in northern cattle) after the young ticks have attached themselves to them.

"The idea has often prevailed that the ticks were in some way connected with the disease in northern cattle. Experiments were made to test this theory.

"Southern cattle were placed in the same enclosure with native (northern) cattle, and the ticks picked off the southern cattle as soon as they were large enough to be detected on the skin This prevented any ticks from maturing and infecting the pasture with eggs, and hence prevented any ticks from infecting native cattle subsequently. At the same time, in another enclosure, the ticks were left on the southern cattle. The natives in the latter field died of Texas fever, those in the former showed no signs of disease.

"Fields were prepared. 1. With southern cattle with ticks. 2. With southern cattle from which the ticks had been removed. 3. One over which the removed ticks were alone scattered. In field number one two natives died. In the tick field one animal died and others said to have been ill. In the third field where no ticks were on the animals none died. These results lead to the following conclusions:

"1. *Texas fever is not caused by bacteria.* Its nature can not be understood by supposing a simple transfer of bacteria from southern cattle to pastures and trom pastures to northern cattle.

"2. The cause is very probably a protozoon with a more complex life history, living for a time within the red corpuscule of infested animals.

"3. Southern cattle without ticks cannot infect a pasture.

"4. Ticks alone scattered on a pasture will produce the disease."

*How the Disease is Transferred from Cattle to Cattle by Ticks.* "There seemed but one inference to be drawn from the facts, that is, *that the young ticks are in some way directly associated with the appearance of the disease.* It requires from forty to sixty days for the matured ticks to drop from the southern cattle and the eggs laid by them to develop into young ticks. The period of the incubation of the disease is explained without any difficulty by the lite history of the tick.

"*The Question was Solved, Experimentally, in the Following Manner:*—Eggs laid by ticks were artificially hatched. The young ticks were placed on four different animals which were kept away from infected enclosures. Of these four, two died of Texas fever, as determined by a careful post-mortem examination. The other two became very ill. In all of them the germs were observed in the blood."

On its face the above statement of apparent facts certainly look very conclusive, but the careful reader, especially if one previously acquainted with the character of other so-called investigations from the same source, will at once notice certain serious discrepancies and certain striking loopholes in the chain of evidence, which a due regard for scientific exactness cannot well overlook, and they should not be overlooked. They are especially called to the attention of the reviewer of this work in *Lancet* and the editor of the *Veterinary Journal*, as well as to that of scientific investigators in general.

They are:

1st. The contradiction between doubt and certainty. In the second conclusion it says, "the cause is very *probably* a protozoön, and the tick is the transmitter of the protozoön." In another place there was no probability about it, for "the question was solved experimentally" in the manner quoted, by placing artificially hatched ticks (from the ova) on northern cattle.

If it was solved it could no longer be problematical. In conclusion "1," it says: "Texas fever is a disease not caused by bacteria." In another place it says: "In one of them (the cattle) the germs were observed in the blood." Perhaps the difference between "germs and protozoa" is immaterial, and I am but making a play on words. The whole story minutely betrays uncertainty of his position on the part of the writer. In no sense have we a particle of true scientific evidence, such as we all have a right to expect of one another in such matters. *We have no illustration of any organism whatever.* Not a plate culture was made from the blood or tissues of any of these animals, nor even do we have a particle of evidence that bouillon culture was attempted.

No mention is made of the inoculation of any kind of small animals (a favorite procedure with these investigators when convenient) either with blood or tissues of the sick or dead cattle. (It is self-evident that cattle under such immediate control could have been killed in time to avoid any post-mortal pollutions). No mention is made of attempts at transmission with the blood or tissues of the diseased cattle, in cattle themselves, a most imperative test experiment in such cases, which should, it would seem, naturally and at once suggest itself to any investigator under the circumstances. In other words: If the protozoa are in the blood of cattle diseased with a specific disease, and the protozoa are the hypothetical cause of the disease, the best way and only way to that question would be:

1st. By plate cultures to assure one's-self that no developable germ, in or on every available media was in that blood.

2d. A similar system of isolation by the use of small animals (the zoospore might fortunately kill in that case, which would be a valuable link in the chain of evidence).

3d. This being done to prove indeed that it was the zoospore (and not something el e in the tick), the inoculation of healthy northern cattle with blood known to contain the zoospore, was absolutely indicated.*

4th. As anthrax can be transmitted by flies, mosquitoes, etc., and as yellow fever is claimed to have been transmitted by the latter, a kindred disease to southern cattle plague (as I have shown in a former edition of this work), why were not the old ticks, with their soiled probosces, placed on healthy northern cattle ?

Had these things been done and fallen negative, they certainly would have been mentioned.

Again, and of equal importance: The "protozoa" are claimed to be the cause sufficiens, the inficiens proper, transmitted, and the tick asserted to be mediator of transmission ; the virus.

Where do the parent ticks obtain the protozoa? Naturally from the blood of the southern cattle ! Then the protozoa should have been as easily demonstrated in sinews from the inside, the blood of the ticks, as in the blood of cattle, but we find no mention of the examination of the ticks in this direction, nor of plate cultures made of their blood.

The protozoa in some way get into the ova and develop up with the young tick ; the ova were in the possession of these men, the protozoa is claimed to be easily colored; why have we no mention of attempts then being made to demonstrate these things in the ova ? Why not in the mates of the young, artificially hatched ticks, from the ova, when they were put on cattle ?

With all due respect to the editors of the *Lancet* and *Veterinary Journal*, I think all unbiased and competent readers must at once admit that they have accepted, and in some measure endorsed the "fishiest yarn" yet promulgated in the name of scientific investigation. A "yarn" absolutely without a scintilla of exact support, except the mere fact of infection, which no one denies, and a second fact, that infection may be in some way connected with ticks.

Let us analyze this tick question a little closer, for it is undoubtedly enchantingly interesting.

So far as they can be hypothecated from the statement of the agricultural department we have these conditions to consider:

* In the report of 1891, just out, it says : "Lest your blood from diseased animals was injected directly into the jugular vein of two healthy animals, subsequent examination indicated a mild attack of Texas fever, but the result was not positive enough. This year the blood of diseased animals was injected into the jugular vein of three healthy animals, and in all a very acute form of Texas fever was produced within four or five days. One died, the others recovered. There can be no doubt that the disease is inoculable from the blood of one animal to another, when the germs are transferred quickly." P. 133. Again, there is no record of plate culture, or a proper examination of the blood.

1st. The mother ticks from Texas are of and in themselves homeless. (It will be remembered that the test to prove that they were was neglected).

2d. They must suck the protozoa from the blood of the diseased cattle.

3d. They drop on the ground, when mature, from the diseased cattle, lay their ova and die.

4th. The protozoa is kind enough to crawl from the intestines of the mother tick, though it walls and penetrate the membrane of the ovum and develop up with the young tick, without injuring either ovum or embryo, then crawl into the intestines and stomach of the young tick.

5th. The young tick, with its protozoic accompaniment, crawls up the legs and onto the bodies of convenient northern cattle, and what does it do? Contracts its abdomen and through its proboscis injects the northern cattle. (It has never been near southern cattle according to the story).

6th. Do the protozoa crawl out of the intestines of the mother tick, and attach themselves externally to the ova and hold on and pass out of the northern tick, and still hold on to the embryo, and are carried by it onto the cattle and then crawl off and into the blood vessels of the cattle through their hides?

These six postulates may appear ridiculous, and I believe they are, to the reader, but in no other way possible can the "sporozoon-tick" hypothesis of the government investigators be explained so far as I can comprehend their statements. As "sporozoa," or anything akin to them, have only been found in malarial disease, and as the essential differentiating characteristics of all malarial diseases is local infection with the absolute impossibility of transmission to other individuals, or of the inficiens to other localities by the sick individual thus far in their history, it looks as if this story from Washington must be taken *cum grano salis giganticus*, and valued on a par with the balance of the testimony from the same source; though there is an error of truth in the tick as will be conclusively shown later on.

As I have taken especial pains to show by quotations from previous announcements and by the universal testimony on those announcements, nothing milder than the Scotch verdict of "not proven" is justifiable in this case.

EXTRACTS.

Extracts from a paper read before the Twenty-seventh Annual Meeting of the United States Veterinary Medical Association, at Chicago, September 17th, 1890, and published in THE JOURNAL OF COMPARATIVE MEDICINE AND VETERINARY ARCHIVES, October, 1890. Pp. 542–3.

"We will now turn our attention for a few minutes to the subject of Texas fever. While this disease, as you know, is allied by its characters to the bacterial fevers, it has certain marked peculiarities, which have caused it to be regarded as the most mysterious malady which remains for modern science to investigate. For four years we have been giving the disease very close attention and study, and our results have been unexpected to ourselves, and so different from those reached by other investigators, that we have not pressed our conclusions until we had taken every precaution to confirm them.

"I am prepared now to say that Texas fever is not a bacterial disease. There are no peculiar bacteria to be found in the blood, spleen, liver, or other organs; and as a rule the blood and tissues are free from even the common septic bacteria when examined immediately after the death of the affected animals. All the illustrations which have been published showing preparations of blood from Texas fever animals swarming with bacteria, and sections of the tissues showing the same micro-organisms, are misleading, and may be put aside as of no value to the student of this disease. The blood and tissues do not present such appearances when properly examined.

"Texas fever belongs to the malarial type of diseases. The germ resembles the malarial organisms of Taveran. It is found within the red corpuscles of the blood, and may be demonstrated in them before the death of the animal. We have not been able to cultivate it, and do not believe that it can be cultivated by any of the methods now in use by bacteriologists.

"We have studied outbreaks that were caused by cattle from the tide-water section of Virginia, from North Carolina and from Texas, and in every case we found the same micro-organism present in the blood. In some cases almost every corpuscle would be invaded. These parasites rapidly destroy the corpuscle, and the number of these soon falls to one-half, one-third, one-fourth, or even one-fifth of what is found in health. This destruction of the corpuscles, of course, accounts for the excretion of the red coloring matter in the urine.

"The possibility of the infection of pastures by ticks has also been demonstrated. For two years we have picked the ticks from southern cattle and scattered them over experimental pastures, and we have found that susceptible cattle would contract the fever from these pastures as readily as from those upon which the southern cattle themselves had been placed. It is a fact, therefore, that ticks may infect pastures without the presence of southern cattle. Whether southern cattle can infect pastures without the presence of ticks is still an open question. We had an experiment planned to settle this problem the present summer, but it failed by the unexpected appearance of the ticks upon this lot of cattle which we had supposed were protected from them.

"The main results of our Texas fever investigations may, therefore, be summed up as follows:

"1. Texas fever belongs to the malarial group of diseases.

"2. It is caused by small micro-organisms which are found in the red blood corpuscles, and which are not bacteria, but bodies of the nature of the malarial germs of Taveran.

"3. Pastures may be infected by ticks from the bodies of southern cattle."

Bold words those in the light of the investigations previously published in Nebraska.

FORMER INVESTIGATIONS FROM THE SAME SOURCE.

Any one who reads the amount of the investigation of one of the sub-divisions of the agricultural department as published in the reports lately referred to, and in the spring of 1891, would scarcely realize that the chief of that division had published quite positive testimony as to a specific germ in Texas fever many years before. One would naturally think that a spirit of common honesty, both as a public servant of a great nation, as well as in obligation to scientific investigators the world over, would have induced the Chief of the Bureau of Animal Industry to have especially admitted that those investigations were erroneous. Of course it may be said that the assertion that "Texas fever is not caused by bacteria," is sufficient, but to my mind that is not enough under the circumstances. Some allusion should have been made to the previously published assertions, which I now quote merely to show that these same investigators, or the chief of them, once did assert "bacteria" to be in the tissues of Texas fever diseased cattle, and that he was in the same maze of uncertainty as to their value as at present regarding the "protozoa;" at one time asserting the cause discovered, and another, not being quite so sure on the matter.

The following quotations are taken from the Report of the Department of Agriculture, 1883, on the

"ESSENTIAL NATURE OF TEXAS FEVER."

"If it had not been for our private laboratory, where our microscopical apparatus and reagents could be used to advantage and where we had fitted up an incubator, imperfect for many purposes, it is true, but still one in which cultivation could be made very successfully, the scientific investigations for the year with this disease must of necessity have been a failure, as such investigations have so generally been in the past," p. 33.

Now, let us be honest and unpredjudiced; what do we understand the government investigator to mean by those words? Can we place any other meaning on them than that he considered his laboratory work, in the investigation of Texas fever, to have been successful, for does he not almost positively compose it in that way with all previous investigations?

"August 1, 1882, I was successful in filling and sealing a number of tubes from the spleen of a diseased cow which had just died. August 7, one of these vacuum tubes was examined microscopically. No decomposition whatever had occurred. Two cultivation tubes were infected with some of this pulp as soon as the tubes were opened; one was found to be a pure cultivation of dipplococci," the other was impure, p. 35. "Two tubes containing splenic pulp were in excellent condition. Both contained the dipplococci found in a former case."

"In 1880, two inoculations were made with splenic pulp, one of which produced a very severe case of the disease, the second was milder, though accompanied with great elevation of temperature," p. 36. Some other experiments failed with the same material, but on "the 3d of October, I obtained some foul material by killing a cow in the last stages of the diseases, and in the afternoon we inoculated a cow in the side of the neck with the splenic pulp obtained in the morning. This animal died in the night of October 16. * * * In this experiment we have a very evident confirmation of my former investigations. The disease is then inoculable and the splenic pulp is the material which contains the virus. Out of five inoculations with this material, we have produced three plain cases of the disease. "Inoculations were also made with the cultivated micrococci." They proved negative. "They do not show that our dipplococcus is the cause of the disease, nor do they show that it is not," pp. 37, 38.

Is it not strikingly singular in the face of the last of the quotations that the chief of the Bureau of Animal Industry should have previously said, in the same report, "the scientific investigations for the year with this disease must have been a failure, had it not been for our private laboratory, as such investigations have so generally been in the past?" In one place he certainly claims success and in another admits failure to prove his position.

Does it not seem natural that in resuming those investigations in 1888 or 1889, some attempts at similar inoculations should have been made by the same investigator to prove or disprove the observations of previous years!

Does not the results of those year's investigations intimate that some bacteria were present in the tissues of the animals?

Does not the question naturally suggest itself, *were* they not afraid of proving too much if they experimented with such tissues later on? and that is why we find no mention of such investigations!

*To be Continued.*

